# Effect of PCL nanofiber mats coated with chitosan microcapsules containing cinnamon essential oil for wound healing

**DOI:** 10.1186/s12906-023-03905-0

**Published:** 2023-03-18

**Authors:** Mahmoud Osanloo, Fariba Noori, Alireza Tavassoli, Mohammad Reza Ataollahi, Ali Davoodi, Morteza Seifalah-Zade, Ali Taghinezhad, Narges Fereydouni, Arash Goodarzi

**Affiliations:** 1 Department of Medical Nanotechnology, School of Advanced Technologies in Medicine, Fasa University of Medical Sciences, Fasa, Iran; 2 Department of Tissue Engineering, School of Medicine, Fasa University of Medical Sciences, Fasa, Iran; 3 Department of Pathology, School of Medicine, Fasa University of Medical Sciences, Fasa, Iran; 4 School of Medicine, Fasa University of Medical Sciences, Fasa, Iran; 5 Noncommunicable Diseases Research Center (NCDRC), Fasa University of Medical Sciences, Fasa, Iran

**Keywords:** Wound healing, Polycaprolactone (PCL), Chitosan microcapsule (µCS), Cinnamon (Cinnamomum zeylanicum (CiZ)), Ion gelation, Electrospinning

## Abstract

**Introduction:**

Cinnamon is one of the most common spices that has been studied for its anti-inflammatory, antioxidant, and antibacterial properties in wound healing. The purpose of this study was to evaluate the effectiveness of polycaprolactone nanofiber mats coated with chitosan microcapsules loaded with cinnamon essential oil in wound healing.

**Material and methods:**

For this purpose, chitosan microcapsules containing cinnamon essential oil (µCS-CiZ) were prepared by ion gelation and PCL nanofibers by electrospinning. The size of the µCS-CiZ and the morphology of nanofibers were evaluated by DLS and FESEM methods. In order to evaluate wound healing, 48 rats in 4 groups of Control, µCS-CiZ, PCL, and PCL + µCS-CiZ and were examined on days 7, 14, and 21 in terms of macroscopy (wound closure rate) and histology (edema, inflammation, vascularity, fibrotic tissue, and re-epithelialization).

**Results:**

The particle size of the µCS-CiZ and the diameter of the nanofibers were estimated at about 6.33 ± 1.27 μm and 228 ± 33 nm, respectively. On day 21, both µCS-CiZ and PCL groups showed a significant decrease in wound size compared to the control group (*P* < *0.001*). The PCL + µCS-CiZ group also showed a significant decrease compared to the µCS-CiZ (*P* < *0.05*) and PCL groups (*P* < *0.05*). Histological results showed further reduction of edema, inflammation, and vascularity in granulation tissue and appearance of moderate to marked fibrotic tissue in PCL + µCS-CiZ group compared with the other groups.

**Conclusion:**

The results of the study showed that the combined use of PCL + µCS-CiZ indicates a synergistic effect on improving wound healing.

## Introduction

Cinnamon is the bark of trees of genus Cinnamomum and is one the most common spices used worldwide especially in Asian ones. There are two main Cinnamomum species; *Cinnamomum zeylanicum* (CiZ) and *Cinnamomum cassia* (CC); the latter is known as *Cinnamomum aromaticum* or Chinese cinnamon. Nearly all parts of Cinnamon tree like leaves, bark, flowers, roots, and fruits are beneficial in medicinal and culinary utilizations. The essential oils obtained from different part of Cinnamon tree vary noticeably in chemical compositions which show different medicinal and pharmacological properties [1]. The bark, leaf, and root mostly possess cinnamaldehyde, eugenol, and camphor, respectively which have the same hydrocarbon backbones with various derivatives [2]. Therefore, this chemical diversity is likely the reason for medicinal and pharmacological benefits which highlights its worthiness to the different industries [3].

*Cinnamomum zeylanicum* (CiZ), also known as Ceylon cinnamon or true cinnamon, originates from Sri-Lanka and India [3]. Almost 83% of the constituents of cinnamon are three essential oils extracted from the bark of the CiZ tree, cinnamaldehyde, eugenol and linalool [4], of which cinnamaldehyde accounts for about 50–63% of the total bark oil [5, 6].

One of the important differences between chemical composition of CiZ and CC is their coumarin content [7]. The level of coumarins in CC seems to be considerably high, which contains approximately 2.1–4.4 gr per kg of CC powder [8]. This means that one teaspoon of CC powder contains about 5.8–12.1 mg of coumarin, which is extremely higher than the Tolerable Daily Intake standards for coumarin reported by European Food Safety Authority (EFSA) [8, 9]. Coumarin is one of the most potential carcinogenic, anticoagulant, and hepato-toxic materials whose underlying mechanisms are yet to be well defined [9]. Consequently, the European Food Safety Authority (EFSA) has warned of health risks from regular consumption of CC powder due to its coumarin content [10].

There are several studies indicating numerous advantageous health effects of CiZ, including anti-tumor, anti-inflammatory, cardio-protective, anti-microbial, and anti-fungal properties [11]. Another important feature of CiZ is wound healing, which has been studied in the form of extracts and essential oils that may be formulated in different methods. Farahpour co-workers (2012) examined wound healing effect of aqueous and ethanolic extract of Ceylon cinnamon in an animal excision wound model. They showed significant wound enclosure rate and epithelialization in cinnamon groups compared to control and placebo groups [12, 13]. Han co-workers (2017) applied CiZ essential oil of bark (CBEO) on primary human neonatal fibroblast cells, and investigated the effect of CBEO on inflammatory and remodeling biomarkers. They showed that CBEO has intense anti-proliferative, anti-inflammatory, anti-remodeling effect and potency to modulate and alter signaling pathways [14].

Therefore, new technologies have developed opportunities to improve the stability, release, quality increase, and effectiveness of natural products. Electrospinning is a method of producing nanofibers from multiple polymer solutions under high-voltage electrical field [15]. This technique is used to produce large-scale complicated structures through different strategies such as multiple-jet [16] and nozzle-less electrospinning [17] with biomedical, drug delivery, and advanced composite properties [18–22]. Nanofibers have particular characteristics, for example high surface to volume ratio, high porosity, inter-connected porous networks, and flexible function which are widely used in medical applications [23–25]. Polycaprolactone (PCL) is one of synthetic polymers, extensively applied for biomedical usage, which is about slow biological degradation and biocompatible properties. The combination of PCL characteristics and individual nanofiber structure properties could lead to a promising scaffold for diverse applications [26–28].

Gosh co-workers (2013) synthesized stable and fine CiZ oil micro-emulsions (CMF4) droplets of very small size of about 6 nm, using non-ionic surfactant Tween-20 and water. CMF4 represented no erythema and anti-bacterial activity and improved wound healing process in rats [29]. Salehi co-workers (2019) fabricated cinnamon loaded into polycaprolactone/gelatin (cin/PCL/Gel) nanofibers in order to improve wound healing. They demonstrated that PCL/Gel 5% cinnamon showed the best wound closure of about 98% compared the other groups after 14 days [30]. Kossyvaki co-workers (2020) also fabricated PVP/keratin electrospun fibers containing cinnamon essential oil UVB burn model. This study was conducted to apply chitosan microparticles containing *Cinnamomum zeylanicum* essential oils to conserve EO stability and effectiveness besides PCL nanofibers to develop an extracellular matrix (ECM)-like scaffold to evaluate wound healing [31].

## Material & methods

### Materials

The materials were purchased as follows: polycaprolactone (PCL) (Sigma–Aldrich, Germany), chitosan (Easter Holding Group, China, MW: 100 KDa, deacetylaton degree: 93%), Cinnamomum zeylanicum (CiZ) essential oil (Zardband Pharmaceutcals Co, Iran), tripolyphosphate (TPP), tween 80, tween 20, Hexafluoro-2-propanol (HFIP), hematoxylin and eosin stain (Merck, Germany), glacial acetic acid and ethanol (> 99.7%, Dr. Mojallali, Iran), and Masson’s trichrome staining kit (Asiapajohesh, Iran).

### PCL fabrication

Electrospun PCL NFs were prepared as previously described [32], with slight modifications. The PCL solution was prepared by dissolving the granules in HFIP (15% w/v) at room temperature overnight. The prepared polymer was loaded into a 10 mL syringe with stainless steel needle (22 gauge) connected to 17 kV DC voltage in an electrospinning apparatus (Fnm co. Ltd., Tehran, Iran). The polymer solution was injected with a feed rate of 0.5 mL/h at the distance of 140 mm from the needle tip to collector rotating at a speed of 100 rpm. For easy separation of formed NFs on the collector, an aluminum foil was wrapped on it.

### Surface morphology analysis

The surface morphology of polycaprolactone nanofibers was analyzed using field emission scanning electron microscopy (FESEM) (MIRA3, TESCAN Co, Czech). The images were taken after the gold plating process at an acceleration voltage of 20 kV (Q150R ES, Quorum Technologies, UK). Then, the average diameter of nanofibers was measured using ImageJ (National Institute of Health, USA) software with a sample size of 100 nanofibers.

### Synthesis of µCS-CiZ

Chitosan microcapsules (µCS) containing CiZ essential oils (µCS-CiZ) were prepared as previously published [33]. Briefly, chitosan solution was prepared by dissolving chitosan (1.5 w/v) in a dilute solution of acetic acid (1% v/v). The chitosan solution, tween 80 (2% w/v), tween 20 (8% w/v) was mixed to form a homogeneous solution and then the essential oil (1% w/v) was added for 5 min and 1500 rpm. Finally, aqueous solution of TPP (0.3% w/v) was drop-wisely added and stirred for another 40 min and left for few days to increase the viscosity and form a gel.

### Size measurement of µCS-CiZ

Particle size and particle size distribution of µCS-CiZ were evaluated using dynamic light scattering (DLS, scatteroscope-I, K-ONE, Korea). d50 (d: median diameter of particles at 50 cumulative percent) as reported by the DLS instrument was considered as particle size and particle size distribution was calculated using the following equation.

$$particle\;size\;distribution\;=\;\sqrt{d75}\;\div d25$$

### FT-IR

FT-IR (Nicolet iS10 FT-IR spectrometer, USA) was used to confirm CiZ loading in the chitosan microcapsules. The FT-IR spectra of CiZ, chitosan, tween 20, tween 80, and microcapsules without CiZ were recorded in wavenumber range of 400–4000 cm^−1^, using KBr pellets.

### In vivo study

In this study, 48 male Wistar rats, which were about 2 months old and weighed 200–250 g, were obtained from animal house of Fasa medical school and used as wound healing model. The rats were kept in polystyrene cages according to the rules of keeping and observing animal rights, with a light cycle of 12 h, standard temperature conditions (25 ± 2 °C), and humidity and free access to food and water. All procedures and experiments involving animals were approved by Bioethics Committee of Fasa University of Medical Sciences (Ethics code: IR.FUMS.REC.1400.070) performed in accordance with the guideline for the care and use of laboratory animals in Iran. All methods are reported in accordance with ARRIVE guidelines.

Prior to the experiments, the cages and chambers were exposed to ultraviolet radiation for 24 h and disinfected using ditol to create a pathogen-free environment. The rats were anesthetized by intraperitoneal injection of ketamine-xylzine (70:30). Then, the animal's back hair was shaved and thoroughly cleaned on the wound area. The expected wound area was disinfected with 10% Betadine solution. Then, using a stencil ruler, a 2 × 2 cm wound was created by a full-thickness surgical razor. Finally, the treatments were applied directly to the newly created wound at the same time of surgery.

### Study groups

Rats were randomly divided into four groups. In each of these groups, there were 4 rats for the study on days 7, 14 and 21. The study groups were studied as follows:

- Control group (wound washing with normal saline and bandage with common sterile gas)
- The rats treated with chitosan microcapsules containing cinnamon essential oil (µCS-CiZ group)
- The rats treated with nanofibers (PCL group)
- The rats treated with microcapsules + nanofibers (PCL + µCS-CiZ group)

The same amount of 2.5 ml of microcapsules and 2.5 × 2.5 cm of nanofibers were used for the experimental groups. This value was selected for the nanofibers based on the size of the wound and for the microcapsules to the extent that it can cover the entire wound. Then, they were kept in separate cages in a special room until the end of the study.

### Macroscopic assessment of the wounds

Wound healing rate was assessed by measuring the length and width of the wound with a caliper on days 7, 14, and 21. Wound closure was performed in different groups with 4 repetitions and the results were reported as mean ± standard deviation.

### Histology study

After sacrificing the animals by CO2 euthanasia on day 21, about 2 mm of the area around the wound was removed with scissors and forceps in full-thickness. Then, the wound tissue was fixed in 10% formalin solution, dehydrated in ascending grade of alcohols, embedded in paraffin, sectioned and mounted on the slides for Hematoxylin–Eosin and Masson’s Trichrome staining and further histological examinations. Qualitative scoring was performed according to Table 1.

**Table 1:** Histopathological scoring of the wound area according to the repairing process

No	Score	Granulation tissue			Fibrotic tissue	Masson's trichrome staining
		Edema	Vascularity	Inflammation		
1	0	Not seen			Not seen	Not seen
2	0–1	Slight			Slight	Slight
3	1	Mild			Mild	Mild
4	1–2	Mild to moderate			Mild to moderate	Mild to moderate
5	2	Moderate			Moderate	Moderate
6	2–3	Moderate to marked			Moderate to marked	Moderate to marked
7	3	Marked			Marked	Marked
8	3–4	Moderately marked			Moderately marked	Moderately marked
9	4	Very marked			Very marked	Very marked

### Statistical analysis

Statistical analyzes were performed using GraghPad Prism 6.0 software. The normality of the data was measured by Kolmogorov–Smirnov test. One-way ANOVA and Tukey post hoc test was performed to compare the mean ± standard deviation (SD) of more than two groups (wound size and re-epithelialization). The edema, inflammation, vascularity, fibrosis tissue, and Masson's trichrome staining severity were analyzed by Kruskal–Wallis test and Mann–Whitney U test was used to determine significance among the groups. Significance level in all analyzes was considered less than 0.05 (*p* < 0.05).

## Results

### Characterization of nanofibers and µCS-CiZ

**Fig. 1:**
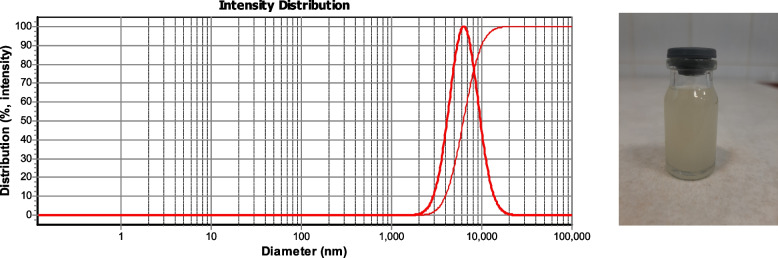
Morphology and DLS of chitosan microcapsules containing cinnamon essential oil

Figure 1 showed that the particle size of µCS-CiZ was 6.33 µm with distribution of 1.27 µm.

### Nanofiber morphology

**Fig. 2:**
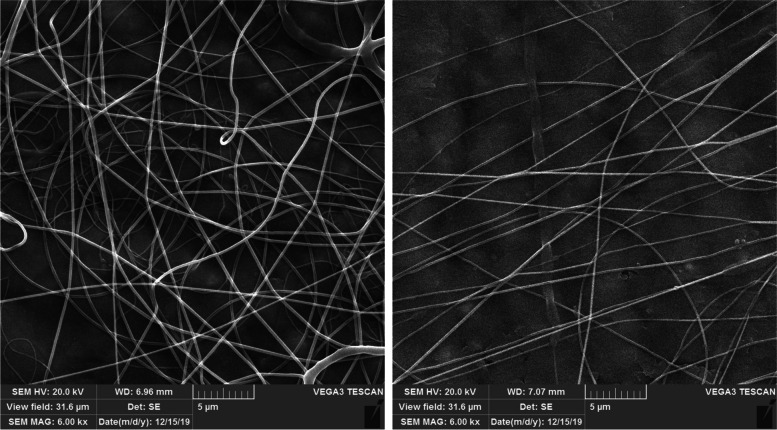
SEM illustration of PCL nanofibers

Figure 2 shows that the PCL nanofibers were formed in the form of an interconnected and non-woven network with a uniform morphology without bead and with numerous pores. By measuring the diameter of 100 nanofibers with ImageJ software, the average diameter of nanofibers was estimated to be about 228 ± 33 nm.

### Fourier transform infrared spectroscopy (FT-IR)

**Fig. 3:**
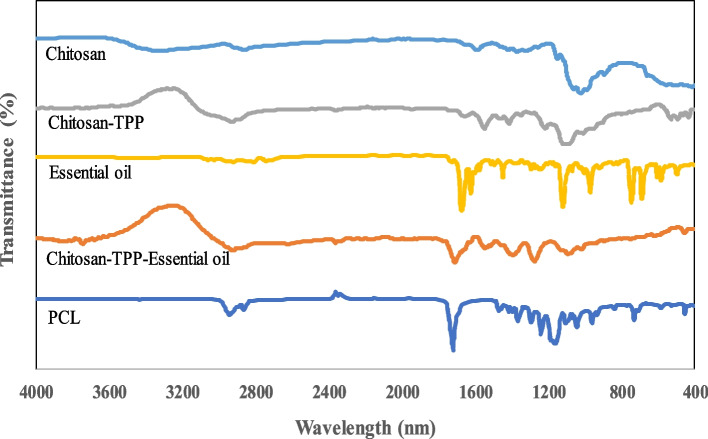
FT-IR of µCS, µCS-CiZ and PCL nanofiber

Figure 3 shows FT-IR spectra of µCS, µCS-TPP, CiZ essential oil, µCS-TPP-CiZ essential oil, and PCL nanofiber. Chitosan powder shows to have specific peaks at different wavenumbers (cm-1) such as 3400–3500 (O–H and N–H (amine I) stretching) and 1550–1650 (N–H (amid II) bending) (33, 35).µCS were formed after addition of an aqueous solution containing TPP into aqueous solution of chitosan, tween 20, and tween 80. Because of electrostatic interactions between phosphoric and ammonium ions of TPP and chitosan, new peak (P = O) appears around 1000–1100 cm^−1^. During the encapsulation process, it leads to slight shift of amide II bending peaks toward shorter wavelengths [33].

Essential oils are usually composed of different ingredients. Characteristic peaks of cinnamon essential oil are mostly seen in the range of 600–1800 cm^−1^. The main peaks of cinnamon essential oil (1727 cm^−1^) correspond to high levels of cinnamaldehyde and aldehyde of saturated fatty acid, and 1679 cm^−1^ and 1626 cm^−1^ are related to stretching vibration of carbonyl aldehyde C = O. To confirm the presence of cinnamon essential oil in the microcapsules, the presence of the most constituent substance is examined. Therefore, the appearance of a peak at 1727 cm^−1^ is related to cinnamaldehyde and confirms the presence of cinnamon essential oil in the formulation of chitosan microcapsules [34].

The polycaprolactone spectrum showed peaks related to -C-H at about 2943, 2894, 2865 cm^−1^ and a very sharp signal band corresponds to the carbonyl group at 1721 cm^−1^. It was also observed that 1470 and 1396 cm^−1^ bands were related to CH2 groups and a signals group around 1292 cm-1 that could be related to asymmetric C–O–C.

### Wound closure

**Fig. 4:**
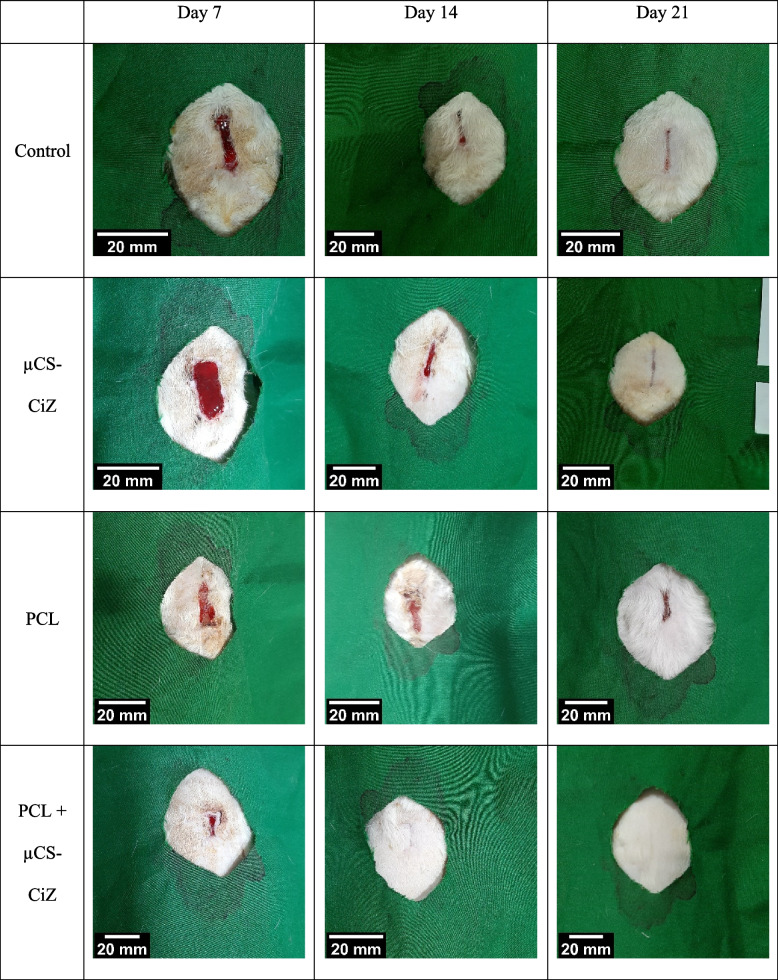
Macroscopic images of wounds of different groups on days 7, 14 and 21. *Control *control group, *µCS-CiZ *chitosan microcapsules containing Cinnamon essential oil group, *PCL *PCL nanofibers group, *PCL* ***+*** *µCS-CiZ *PCL nanofibers coated with chitosan microcapsules containing cinnamon essential oil group

**Fig. 5:**
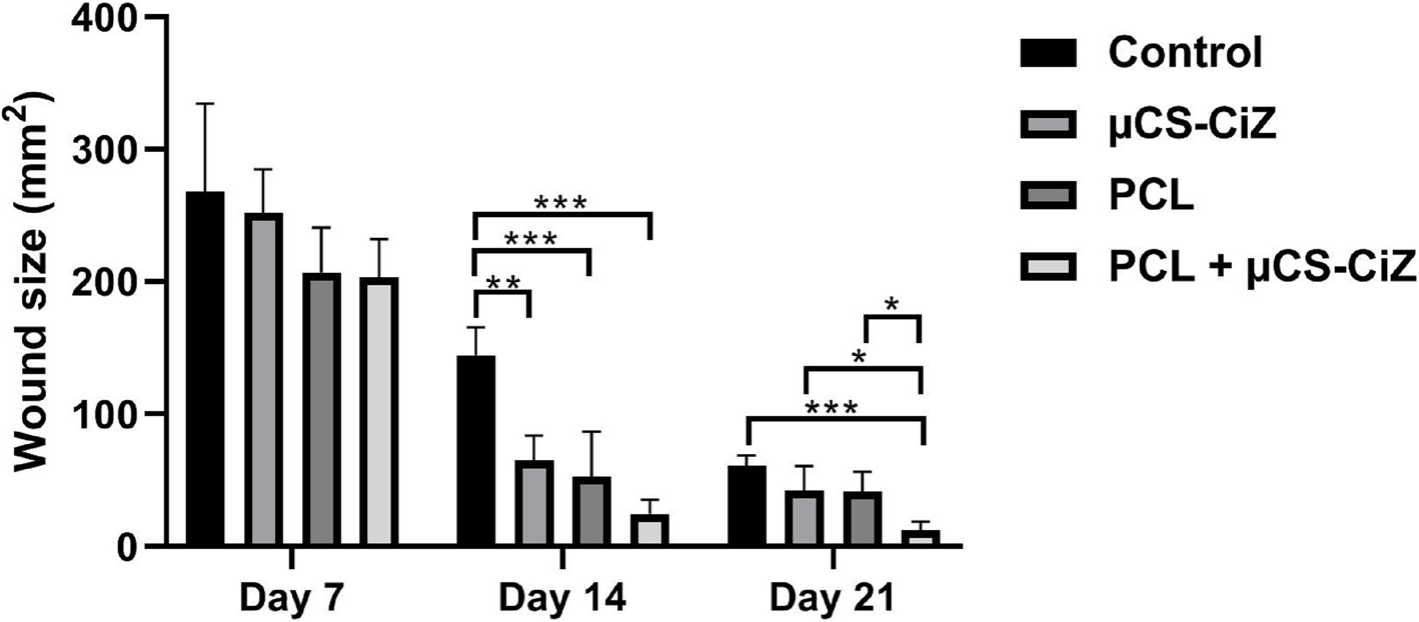
Wound size comparison of control, µCS-CiZ, PCL, and PCL + µCS-CiZ on days 7, 14, and 21, *: *P* < *0.05*, **: *P* < *0.01*, and ***: *P* < *0.001* shows the difference of significance level between the groups. *Control *control group, *µCS-CiZ *chitosan microcapsules containing Cinnamon essential oil group, *PCL *PCL nanofibers group, *PCL + µCS-CiZ *PCL nanofibers coated with chitosan microcapsules containing cinnamon essential oil group

Figure 4 shows macroscopic images of wounds on days 7, 14 and 21 in different groups. Statistical analysis of these images in Fig. 5 and Table 2 shows that the rate of wound closure in different groups on day 7 are not significantly different. On day 14, the µCS-CiZ group showed a significant decrease compared to the control group (*P* < 0.01), the nanofibers group compared to the control group (*P* < *0.001*) and the PCL + µCS-CiZ group compared to the control group (*P* < *0.001*). However, on day 21, the PCL + µCS-CiZ group alone showed a significant decrease compared to the control group (*P* < *0.001*), the PCL + µCS-CiZ group also showed a significant decrease compared to the µCS-CiZ group (*P* < *0.05*) and the nanofibers group (*P* < *0.05*).

**Table 2:** Wound size of four groups on days 7, 14, 21

	**Control**	**µCS-CiZ**	**PCL**	**PCL + µCS-CiZ**
Day 7	267.98 ± 66.41	252.09 ± 32.70	206.68 ± 34.23	203.19 ± 28.96
Day 14	144.68 ± 21.15	65.05 ± 18.89	53.27 ± 33.43	24.51 ± 10.74
Day 21	60.76 ± 8.22	42.26 ± 18.56	41.74 ± 14.90	12.28 ± 6.49

*Control *control group, *µCS-CiZ *chitosan microcapsules containing Cinnamon essential oil group, *PCL *PCL nanofibers group, *PCL* + *µCS-CiZ *PCL nanofibers coated with chitosan microcapsules containing cinnamon essential oil group

### Histological studies

**Fig. 6:**
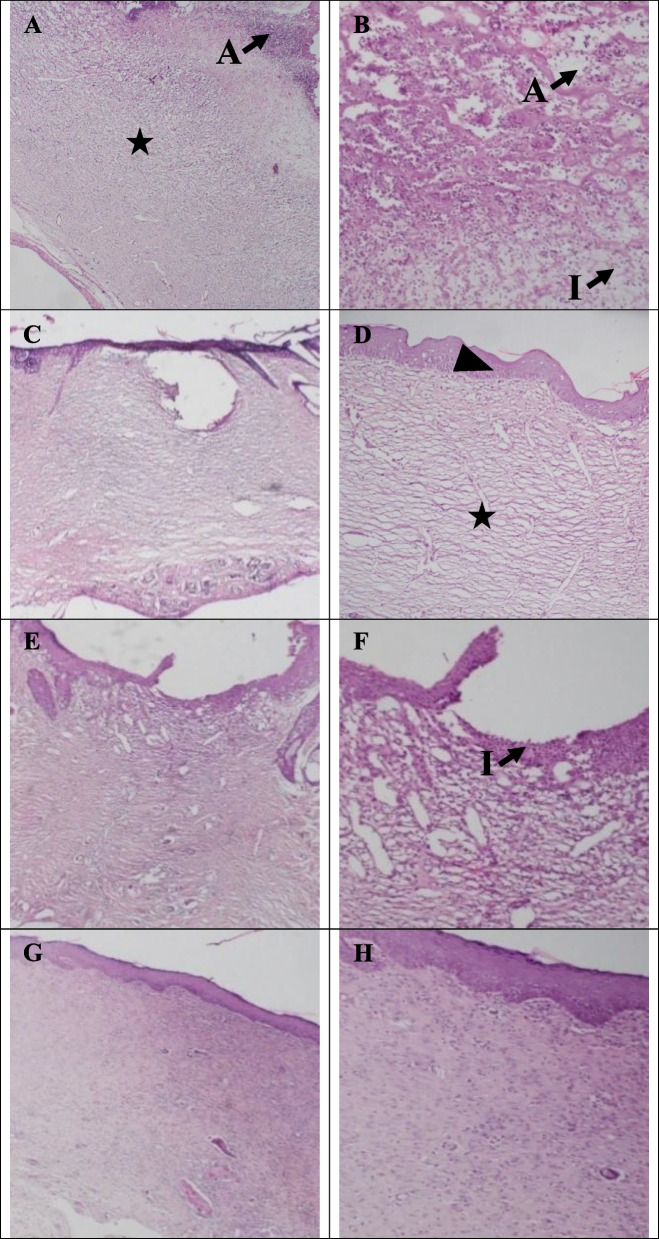
Hematoxylin–Eosin staining of Control group (**A** (40X)-**B** (100X)), µCS-CiZ group (**C** (40X)-**D** (100X)), PCL group (**E **(40X)-**F **(100X)), PCL + µCS-CiZ group (**G **(40X)-H(100X)) on day 21. **Star sign:** granulation tissue, **Cross sign:** fibrotic scar tissue, **Arrow sign with letter I:** inflammation, **Arrow sign with letter** **A**: micro-abscess, **Arrow sign with letter** **E**: edema, and **Head arrow:** re-epithelialization

**Fig. 7:**
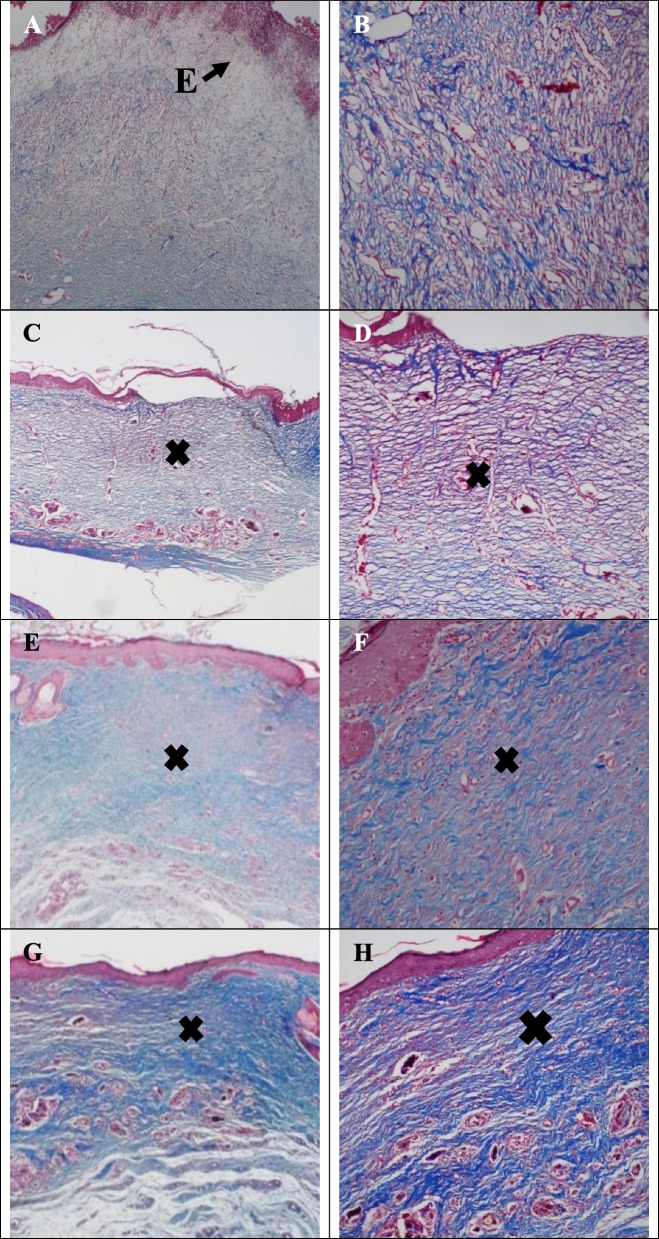
Masson's trichrome of Control group (**A** (40X)-**B** (100X)), µCS-CiZ group (**C** (40X)-**D** (100X)), PCL group (**E **(40X)-F(200X)), PCL + µCS-CiZ group (**G **(40X)-H(100X)) on day 21. **Star sign:** granulation tissue, **Cross sign:** fibrotic scar tissue, **Arrow sign with letter I:** inflammation, **Arrow sign with letter** **A**: micro-abscess, **Arrow sign with letter** **E**: edema, and **Head arrow:** re-epithelialization

In the control group, ulceration in the epidermal layer, loose granulation tissue, micro-abscesses, and moderate to marked infiltration of inflammatory cells in wound area indicated early stages of granulation tissue formation and incomplete healing (Fig. 6A-B). Masson's trichrome staining also showed that granulation tissue does not convert to fibrotic scar tissue in any of the control groups. The light blue color in the wound site is mainly due to protein and fibrin leakage from the vessels of granulation tissue (Fig. 7A-B).

In the µCS-CiZ group, re-epithelialization of the thickness of the epidermal layer is about 17–50% of the adjacent layer. There is primary granulation tissue formation as well as infiltration of inflammatory cells and edema (Fig. 6C-D). Masson's trichrome staining also showed that mild to moderate fibrotic scar tissue fills the subcutaneous wound area (Fig. 7C-D).

In the PCL group, epidermal ulcers, micro-abscesses, and vascular granulation tissue are present with moderate to marked infiltration of inflammatory cells. In this group, epidermal regeneration is not seen in contrast to the µCS-CiZ group, so that the microcapsule seems to play an important role in re-epithelialization (Fig. 6E-F). Masson's trichrome staining also showed moderate color for fibrotic scars (Fig. 7E-F).

In the PCL + µCS-CiZ group, reduction of vascular inflammation and edema in the granulation tissue (Fig. 6G-H) and in Masson's trichrome staining, moderate to high fibrotic ulcer (Fig. 7G-H) implied that the combined effect of µCS-CiZ and PCL displaces more fibrotic scar tissue by the granulation tissue. However, in this group, re-epithelialization does not occur completely and the wound surface is observed in most cases of this group.

### Statistical analysis of histological staining

**Fig. 8:**
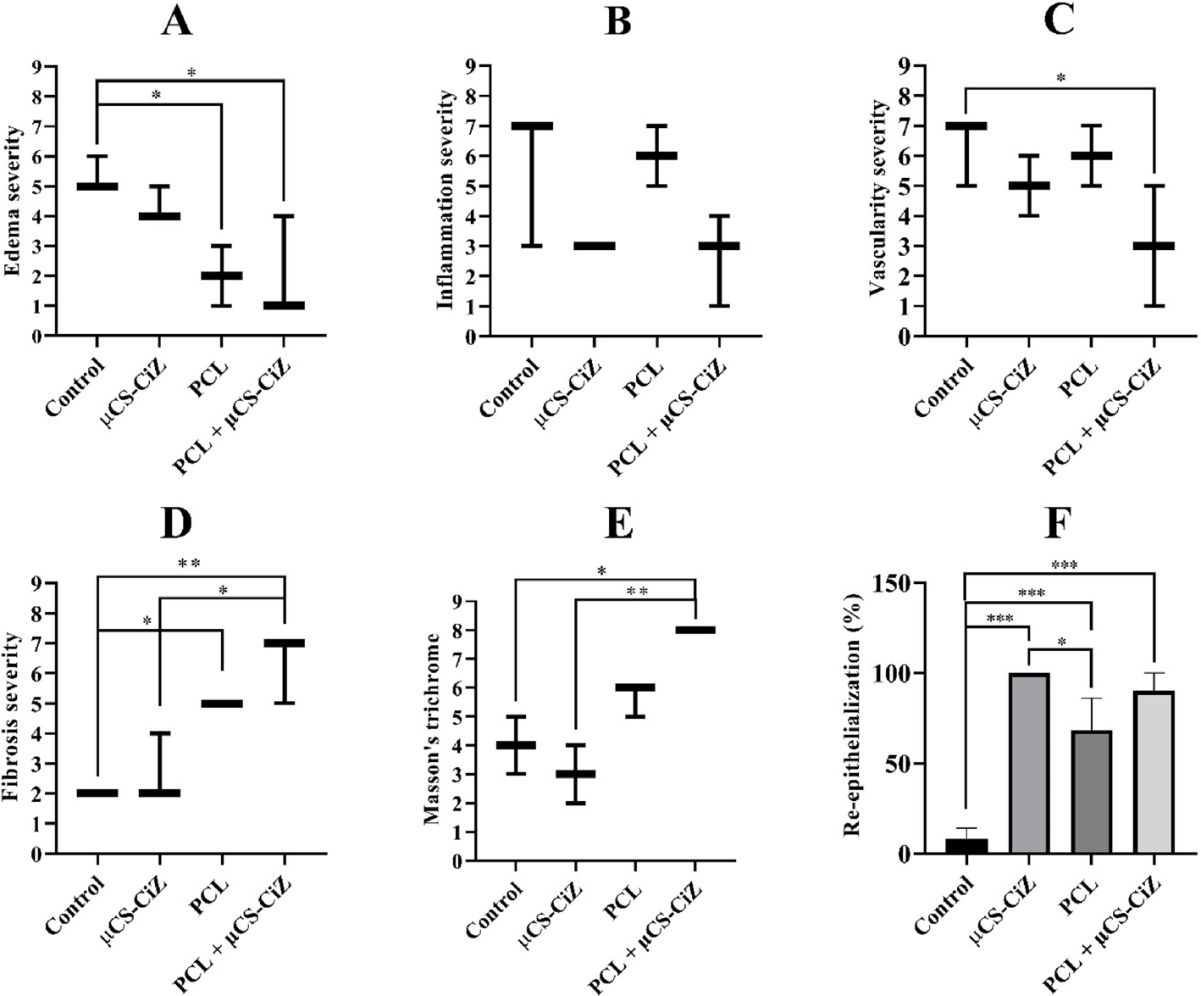
Statistical analysis of histological factors: Edema severity (**A**), Inflammation severity (**B**), Vascularity severity (**C**), Fibrosis severity (**D**), Masson’s trichrome (**E**), Re-epithelialization percentage (**F**). *: *P* < *0.05*, **: *P* < *0.01*, and ***: *P* < *0.001* shows the difference of significance level between the groups. *Control* control group, *µCS-CiZ* chitosan microcapsules containing Cinnamon essential oil group, *PCL* PCL nanofibers group, *PCL + µCS-CiZ *PCL nanofibers coated with chitosan microcapsules containing cinnamon essential oil group

In this study, three parameters of granulation tissue including edema, inflammation, and vascularity were examined separately. Figure 8 showed that edema severity had a significant decrease in the PCL and PCL + µCS-CiZ groups compared to the control group (*P* < *0.05*). Vascularity severity also decreased significantly in the PCL + µCS-CiZ group (*P* < *0.05*). Statistical studies also showed that the PCL + µCS-CiZ group had a significant increase in fibrosis tissue accumulation and replacement with granulation tissue compared to the control group (*P* < *0.01*) and µCS-CiZ group (*P* < 0.05). This result was confirmed by the results of Masson's trichrome staining, as it showed that the PCL + µCS-CiZ group had a significant increase in the collagen filament secretion intensity compared to the control group (*P* < *0.05*) and µCS-CiZ group (*P* < *0.01*). In general, the results showed that PCL + µCS-CiZ could improve the passage of granulation tissue stage and the onset of fibrous tissue secretion significantly faster than the control group.

Statistical results showed that re-epithelialization had a significant increase in µCS-CiZ, PCL, and PCL + µCS-CiZ groups compared to the control group (*P* < *0.001*). In addition, this parameter showed a significant increase in the µCS-CiZ group compared to the PCL group (*P* < *0.05*) (Fig. 8).

## Discussion

This study was performed to evaluate the effects of PCL nanofibers scaffold coated with chitosan microcapsules containing cinnamon essential oil on the healing of full-thickness wounds in an animal model of rats. Inflammation, fibroblast proliferation, collagen deposition, wound contraction, and re-epithelialization are the most important stages of wound healing and have close relationship with each other. Therefore, intervention in any of these stages using beneficial drugs can ultimately promote or inhibit one or all of the recovery stages [35]. Herbal medicines are increasingly used worldwide due to their effectiveness and safety. Cinnamon is a valuable medicinal plant that has multiple healing properties. Recent studies reported the antioxidant activity of cinnamon essential oil [36]. Cinnamon essential oil can inhibit hepatic 3-hydroxy-3-methyl glutaryl CoA reductase (HMG-CoA) activity in mice and lipid peroxidation by increasing the activity of liver antioxidant enzyme [37]. Moreover, having Eugenol content, cinnamon oil has anti-inflammatory property like COX antagonist, which accelerates wound healing [38, 39].

It has been shown that cinnamon extract, whether aqueous or alcoholic, in addition to its specific antioxidant properties, may also have antimicrobial effects, which could be the basis for the wound healing activity of cinnamon [12, 40, 41]. Cinnamaldehyde, a bioactive compound in cinnamon, has significant antibacterial activity against gram-positive and gram-negative bacteria in vitro [42, 43]. In addition, cinnamaldehyde also inhibits the growth of fungi such as yeast, filamentous mold and dermatophytes, and eggs and adult head lice [44]. Therefore, all these reported properties of cinnamon have anti-inflammatory, antioxidant, and antimicrobial properties that promote wound healing, mostly due to the substances contained in its essential oil, especially Eugenol and Cinnamaldehyde.

In this study, chitosan microcapsules containing cinnamon essential oil were used for wound healing. Histological results show that both collagen production and re-epithelialization improved in the µCS-CiZ group alone compared to the control group. Wound dressings are classified as inactive and active according to their role in wound healing. Dressings that only cover wounds are classified as passive dressings while dressings that, in addition to their primary use, heal wounds are considered active dressings [45]. Recently, special attention has been paid to the manufacture of drug-carrying dressings to target different stages of wound healing [46, 47]. For this purpose, several drug carriers have been formulated, among which, polymer particles have attracted a lot of attention due to their new properties and functions compared to conventional drug delivery systems [48]. Degradable and biocompatible chitosan particles are a group of these carriers that can act as drug carriers for wound healing drugs [49]. These particles were used in this study to carry cinnamon essential oil due to its antibacterial, antioxidant and wound healing properties. On the other hand, low solubility and rapid degradability of cinnamon essential oil disrupts its bioavailability in the wound area, which can be preserved by trapping polymer particles [50, 51]. Various studies have also shown that the use of chitosan at the wound site can accelerate wound contraction. The wound area of chitosan-treated animals was significantly lower than untreated groups. Chitosan can be depolymerized and release acetyl glucosamine (N-acetyl-β-D-glucosamine) which promotes fibroblast proliferation and increases collagen deposition at the wound site [52, 53]. Studies have shown that chitosan is able to reduce the number of bacteria, inflammatory factors, and oxidative stress, thereby helping to heal wounds [54, 55].

In this study, polycaprolactone nanofibers were also used as a scaffold to improve wound healing. Histological results show that in the nanofibers group alone, the rate of collagen production and conversion of granulation tissue to fibrotic tissue has improved compared to the control group, but not better than the other groups. New technologies have created opportunities to improve the sustainability, release and increase the quality and effectiveness of natural products. Electrospinning is a method for producing nanofibers from a polymer solution under a strong electric field [15]. This method, through various strategies such as multi-nozzle electrospinning [16] and nozzle-less electrospinning [17], is used to produce complex large-scale structures with biomedical properties, drug delivery systems and advanced composite nanofibers with fillers for biocompatible scaffolds [18–22].

Nanofibers have unique properties widely used in medical applications, such as high surface to volume ratio, high porosity, interconnected porous network, and flexible performance [23–25]. Polycaprolactone (PCL) is a synthetic polymer that is widely used in medical applications due to its slow biodegradation and biocompatibility properties. The combination of PCL (biocompatibility and slow biodegradation) properties with the unique structural properties of nanofibers has led to the creation of a promising substrate for a variety of applications, including medical applications [26–28]. PCL nanofibers can be used as a skin replacement or as a wound dressing. Flexibility to combine with other bioactive substances (such as growth factors, nanoparticles, antimicrobials, anti-inflammatory agents, and wound healing drugs) is another advantage in nanofibers [56–59]. Nanofiber membranes with their barrier-like function are considered as a physical barrier to the penetration of microbes and thus prevent infections. In addition, the pores in nanofiber scaffolds (typically 1 to 10 microns) are small enough to prevent bacteria from infiltrating. In addition, due to their extracellular matrix-like structure, they facilitate and promote cell migration from the outer edges of the wound to the center. Finally, PCL nanofiber membranes provide an effective method for faster wound healing [60].

This study also shows that concomitant use of PCL nanofibers with chitosan microcapsules containing cinnamon essential oil has a synergistic effect on wound healing. Combined use of both scaffolds has caused faster transformation of the granulation to fibrotic tissue and the wound closure faster than the control group, as well as the use of each one separately. These results show that the combined use of microcapsules containing cinnamon essential oil with nanofibers creates greater compatibility.

## Data Availability

All data generated during the current study are available from the corresponding author on reasonable request.
